# Job precarity impacts the mental health of contractual teachers in Morocco: between fatigue and psychological distress

**DOI:** 10.11604/pamj.2024.48.158.43552

**Published:** 2024-08-07

**Authors:** Fatima Bouizzal, Hicham Guider, Merouane El Mourabit, Youssef El Madhi, Moulay Laarbi Ouahidi

**Affiliations:** 1 Laboratory of Biology and Health, Faculty of Science, Ibn Tofail University, P.O. Box 133, Kenitra 14000, Morocco,; 2 Laboratory of Electronic Systems, Information Processing, Mechanics and Energy, Faculty of Science, Ibn Tofail University, Kenitra, Morocco,; 3 Laboratory Education, Environment and Health, Regional Center for Education and Training Professions, Rabat, Morocco

**Keywords:** Psychological distress, fatigue, contractual teachers, general health questionnaire, individual strength checklist

## Abstract

**Introduction:**

since the Ministry of National Education introduced contractual recruitment in 2016, Morocco has faced significant challenges related to the well-being of its contractual teachers. This study investigates the impact of job precarity on the mental health of these teachers, specifically focusing on fatigue and psychological distress.

**Methods:**

we collected responses from 245 contractual teachers across Morocco’s 12 regions, utilizing the Individual Strength Checklist (CIS) to assess fatigue and the General Health Questionnaire (GHQ) for psychological distress. Our findings reveal that teachers' average scores on the CIS (51.7 ± 19.7) and GHQ (12.3 ± 4.6) were notably high, indicating significant job-related stress and emotional suffering.

**Results:**

our study indicates that teachers had very high average scores on the CIS (51.7 ± 19.7) and GHQ (12.3 ± 4.6), suggesting that they experienced considerable job-related stress and emotional distress. Our research revealed that 31% of teachers reported experiencing weariness, while 26% reported experiencing psychological distress. Additionally, out of the individuals who reported experiencing chronic exhaustion, 39% specifically experienced fatigue alone, while 61% experienced both fatigue and psychological discomfort. This suggests a significant association between these two conditions.

**Conclusion:**

the research emphasizes that Moroccan contractual teachers have a shared experience of exhaustion and mental anguish, which is worsened by the uncertainty of their job. Specific interventions are required to address and alleviate these unique effects on teachers' well-being, thereby enhancing the entire educational atmosphere.

## Introduction

Fatigue is a symptom commonly reported by patients in medical consultations, affecting about 20% of the general population, with a higher prevalence in women [1,2]. A variety of factors, from pathological and environmental conditions to psychological and nutritional aspects [3], can influence it, and it is frequently associated with physical and psychiatric disorders, particularly depression [4,5]. In the professional context, the repetitive and demanding nature of work closely links to the experience of fatigue. Teachers, in particular, face unique challenges that exacerbate this phenomenon. Preparing lessons, evaluating and remediating students' learning difficulties, managing the class, and collaborating with colleagues and school administration all require sustained efforts, which, in the absence of effective stress management, can lead to pronounced fatigue and increased psychological distress [6].

Increased stress, a heightened sense of fatigue, doubts about one's abilities, and a decrease in motivation often manifest this distress. In some cases, this may push teachers to consider resigning [7]. Not only can fatigue affect teachers' health and mental well-being, but it can also negatively impact the quality of education they deliver, highlighting the importance of recognizing and addressing these issues [8]. Symptoms of psychological distress, such as anger, irritability, anxiety, and exhaustion, further complicate the situation, contributing to low self-esteem and social isolation [9]. The relationship between fatigue and psychological distress can vary among populations and professions, typically due to a combination of poor mental health and high workloads [10].

Few studies have specifically focused on fatigue and psychological distress among teachers, and no study in Morocco has examined the relationship between these two states. We conducted a questionnaire study on these two conditions among teachers between November 2022 and February 2023. The objectives were: (1) to determine the prevalence of fatigue and psychological distress among contractual teachers in Morocco; and (2) to explore the relationship between fatigue and psychological distress among these teachers.

## Methods

**Study design:** the purpose of this research was to investigate how job precarity affects fatigue and psychological distress in Moroccan contractual teachers. The study is a component of a wider framework that evaluates the psychosocial effects of working conditions in the education sector.

**Setting:** the study was carried out in 12 regions of Morocco, encompassing the country's geographical and demographic variations. The process of recruiting participants and collecting data occurred from November 2022 to February 2023. The exposure periods under consideration encompass the entire duration of the instructors' careers, starting from their initial contractual recruitment.

### Participants

**Eligibility criteria:** the study included contractual teachers aged 30 to 60 who worked in the public sector across Morocco's 12 regions. Exclusion criteria included the absence of a valid employment contract at the time of the study and refusal to consent to participate.

**Selection of participants:** we recruited participants by distributing a call for participation online and on professional platforms specifically for teachers. The selection was carried out to ensure equal representation of the different regions and levels of teaching.

### Data sources/measurement

**Measuring fatigue:** fatigue was measured using the Checklist of Individual Strength (CIS) scale. The working population has validated this scale [11]. It covers four aspects of fatigue: severity, concentration, motivation, and physical activity level. On a seven-point Likert scale, the subject must express, for each statement, how they felt during the last two weeks. High scores indicate high levels of fatigue, reduced concentration and motivation, and low levels of activity. Teachers who obtained a total CIS score >76 were considered probable cases of fatigue [12].

**Measurement of psychological distress:** psychological distress was assessed using the 12-item General Health Questionnaire (GHQ) [13,14]. We designed the GHQ-12 as a screening instrument for minor psychiatric disorders in the general population. Two scoring systems were used: the four-point response scale (0, 1, 2, 3) in the recent version and the traditional method (0, 0, 1, 1). The traditional approach seeks to identify individuals who display significant psychological suffering, thereby categorizing them as likely cases of mild psychiatric disorders. Individuals who obtained a score of four or higher were classified as experiencing psychological discomfort.

**Sociodemographic and health-related variables:** we collected data on the gender, age, educational background, years of experience, teaching level, and health condition (rated as “excellent”, “very good”, “good”, “moderate”, or “poor”) for each instructor.

**Bias:** to mitigate potential sources of bias, we took measures to guarantee the anonymity of participants and employed internationally standardized and acknowledged questionnaires. In addition, we performed an initial analysis to identify and remove any responses that were inconsistent or incomplete.

**Statistical analysis:** we employed principal component analysis (PCA) to validate the variables of the two scales for the Clinical Interview Schedule (CIS) and the General Health Questionnaire (GHQ). In addition, we employed other statistical methods such as Pearson's correlation, the Chi-square test, Student's t-test, analysis of variance, and analysis of covariance.

**Ethical consideration:** the study adhered to ethical standards, ensuring participants' consent and the confidentiality of their data. Ethical approval was obtained from the relevant institutional review board.

## Results

**Participants:** at the start of our study, we identified 300 contractual teachers from 12 Moroccan regions as potentially eligible. After an eligibility examination, we confirmed that 270 teachers were eligible. Of these 270, 25 did not participate in the study for various reasons, including lack of time and disinterest in the subject, leaving 245 participants included in the study. The final analysis included all 245 participants who completed the follow-up. Time constraints and limited internet access were the main reasons for non-participation in completing the questionnaires.

**Descriptive data:** the gender distribution was 45% for men and 55% for women. The mean age was 43.2 years (standard deviation of 11.3). The distribution of teachers according to their level of education reveals that 82% have a bachelor´s level, 14% have a master´s level, and 4% are doctors. Nearly half of the teachers have 10 to 15 years of teaching experience. According to teachers, their health status varies between excellent: 19%; good: 43%; moderate: 32%; and poor: 6%. Table 1 presents the distribution of our sample according to different demographic and health factors.

**Table 1:** distribution of the sample according to sociodemographic variables

Variable	N (%)*
**Age (in years)**	
30 - 40	40 (16)
40 - 50	128 (52)
50 - 60	77 (32)
**Level of education**	
Bachelor’s degree	201 (82)
Master’s degree	35 (14)
PhD	9 (4)
**Teaching level**	
Primary	189 (77)
Secondary	56 (23)
**Seniority**	
5 - 10 years	42 (17)
10 - 15 years	118 (48)
15 years and over	85 (35)
**Health status**	
Excellent	47 (19)
Good	106 (43)
Moderate	78 (32)
Poor	14 (6)

* N (%): Number of participants (percentage)

### Outcome data

**Distribution of fatigue and psychological distress among teachers:** the assessment of fatigue among teachers revealed an average score on the CIS scale of 51.7 with a standard deviation of 19.7, indicating a continuous distribution of fatigue scores ranging from 20 to 140. Similarly, the GHQ measured psychological distress, revealing an average score of 12.3 (standard deviation of 4.6) that ranged from 0 to 36, demonstrating a continuous distribution within our sample of teachers (Table 2).

**Table 2:** comparative analysis of fatigue and psychological distress by age and gender among teachers

Age group	Fatigue percentage	Psychological distress percentage
**From 30 to 40 years**		
Male	45.2%	11.3%
Female	47.3%	11.9%
**40 to 50 years**		
Male	51.3%	12.5%
Female	52.9%	12.8%
**50 to 60 years**		
Male	59.5%	12.9%
Female	60.1%	13.1%

**Psychometrics of the CIS and GHQ-12:** we observed a significant correlation (r = 0.56) between the total scores of the CIS and the GHQ-12, indicating a positive relationship between fatigue and psychological distress. The validity and reliability of the two instruments were confirmed, with a four-factor structure for the CIS replicated, covering severity, concentration, motivation, and physical activity level, demonstrating high internal consistency (α = 0.91-0.95) and good test-retest reliability (r = 0.84 to 0.86). The GHQ demonstrated satisfactory reliability with a Cronbach´s alpha of 0.82. Numerous studies have validated the GHQ tool as a reliable measure of mental health in different populations around the world [15-17].

**Associations of fatigue with demographic and health factors:** a detailed analysis highlighted a slight difference in average fatigue scores between men (56.2; SD 21.5) and women (58.1; SD 23.3), without a strong correlation with age. As indicated in Table 3, older teachers (50-60 years) reported significantly higher levels of fatigue (p < 0.05), and a notable correlation was established between fatigue and the presence of illnesses (p < 0.001), indicating higher fatigue scores among teachers suffering from illnesses.

**Table 3:** associations between levels of fatigue and psychological distress and demographic as well as health factors among education professionals

Factor	Fatigue (CIS)	Psychological distress (GHQ)
**Level of education**		
Bachelor	55.2	12.8
Master's	41.7	11.5
Doctorate	31.5	9.9
**Teaching level**		
Primary	43.2	12.4
Secondary	49.3	13.5
**Seniority**		
5 - 10 years	45.2	14.3
10 - 15 years	51.3	12.5
15 years and over	59.5	11.7
**Health status**		
Excellent	49.3	9.3
Good	51.5	10.5
Moderate	57.2	13.9
Poor	58.3	15.2

CIS: checklist of individual strength; GHQ: general health questionnaire

**Associations of psychological distress with demographic and health factors:** regarding psychological distress, average scores were slightly higher among women (12.9; SD 6.1) compared to men (12.1; SD 4.2), with a positive but very weak correlation with age. Younger teachers (30-40 years old) reported significantly lower levels of psychological distress compared to their older colleagues.

**Association of fatigue and psychological distress:** among participants who expressed prolonged fatigue, 61% also reported experiencing psychological distress, demonstrating a strong correlation between these two states (psychological distress and fatigue).

## Discussion

Our study revealed a significant correlation (r = 0.69) between fatigue and psychological distress among contractual teachers in Morocco, aligning with the initial objectives of the study. This correlation supports the hypothesis that teachers' psychological well-being is influenced by their working conditions [18,19]. The results reflect international trends, as highlighted by Nwoko *et al*. (2023) and Okwaraji *et al*. (2015), showing that the teaching profession is particularly prone to fatigue and psychological distress [20,21].

**Limitations:** the self-reported data collection methodology of this study may introduce response bias. The similar items in the CIS and GHQ may explain some overlap between prolonged fatigue and psychological distress, but principal component analysis (PCA) revealed a separation between the CIS and GHQ items, suggesting two distinct but underlying constructs. Additionally, the specificity of the Moroccan context and the selection of only contractual teachers limit the generalizability of the results, potentially not reflecting the experience of all Moroccan teachers.

**Interpretation:** the interpretation of our results highlights a complex and multifaceted reality of the psychological well-being of contractual teachers in Morocco, revealing a significant correlation between fatigue and psychological distress. Our study enriches this body of knowledge by specifically highlighting the potentially exacerbating effect of contractual status on teachers' psychological experiences. The Moroccan context's uniqueness implies heightened susceptibility to uncertainty and job precarity, highlighting the critical significance of customized support strategies. The separation between the CIS and GHQ items, revealed by our principal component analysis, reinforces the conceptual distinction between fatigue and psychological distress. This confirms the existence of two distinct well-being dimensions, which, although often interconnected, require targeted approaches for their evaluation and management. Kirk *et al*. (1999), in their study on a population of Australian twins, identified a similar separation between fatigue and anxiety/depression, further supporting the specificity of these constructs [22].

The influence of gender on fatigue, more pronounced among women in our sample, as well as the complex relationship between age, education level, and marital status and fatigue and psychological distress, highlights the role of demographic factors as potential modulators of these phenomena. These results align with the work of Hickie *et al*. (1996) and Hardy *et al*. (1997), who also highlighted gender and social status-based differences in the prevalence of fatigue [1,10]. A study on fatigue in the general Norwegian population [2] found a weak association between age and fatigue.

The observed relationship between education level and fatigue is a linear trend toward lower fatigue scores with increasing education level, found in the present study in agreement with data from Loge *et al*. although previous research has shown no effect of age on fatigue [2]. Finally, a holistic approach to teacher support is required due to the strong association between health status and these two psychological dimensions, particularly the impact of illness on the increase in fatigue and psychological distress. This finding, in resonance with Chen *et al*. (1986), prompts a broader reflection on workplace health policies and well-being programs for education professionals [1,3,18,19,23].

**Generalizability:** we must approach the generalization of our findings to other populations or contexts with caution, even though they provide important insights into the effects of fatigue and psychological distress among contractual teachers in Morocco. Differences in the education system, institutional support, and working conditions between countries may influence teachers' well-being experiences. However, this study significantly contributes to the research on teacher well-being, indicating the need for targeted strategies to support this professional population.

## Conclusion

This study emphasizes a notable association between exhaustion and psychological distress among contractual teachers in Morocco, suggesting that employment insecurity has a considerable effect on their mental well-being. The results indicate that a significant fraction of these educators encounter elevated levels of exhaustion and mental anguish, with older and female educators being disproportionately impacted. The research indicates that the lack of stability in contractual work worsens these problems, emphasizing the necessity for specific interventions to assist the well-being of this vulnerable professional group. By implementing comprehensive support solutions, the negative impacts of job precarity can be reduced, leading to improvements in the educational environment and the overall well-being of teachers. Considering the particular circumstances of Moroccan contractual instructors, these findings offer valuable perspectives but should be applied cautiously to other groups. Subsequent investigations should examine similar phenomena in other educational and cultural contexts to formulate solutions that can be universally applied.

### What is known about this topic

- *Mental health challenges in teachers: the teaching profession is associated with higher levels of psychological distress due to job demands and stressors;*
- *Effects of employment precarity: contractual or temporary employment status exacerbates mental health issues, with job insecurity contributing significantly to stress and anxiety;*
- *Link between stress and teacher well-being: job-related stress negatively impacts teachers' well-being, affecting their performance and student outcomes*.

### What this study adds

- *Insights into Morocco's educational sector: offers a focused examination of how contractual employment since 2016 affects Moroccan teachers' mental health;*
- *Data on fatigue and psychological distress: provides quantitative analysis of the prevalence and correlation of fatigue and psychological distress among contractual teachers in Morocco;*
- *Role of social and demographic factors: identifies social and demographic factors influencing mental health among teachers, enriching the understanding of employment conditions' impact on well-being*.
